# Is Sweet Taste Perception Associated with Sweet Food Liking and Intake?

**DOI:** 10.3390/nu9070750

**Published:** 2017-07-14

**Authors:** Shakeela N. Jayasinghe, Rozanne Kruger, Daniel C. I. Walsh, Guojiao Cao, Stacey Rivers, Marilize Richter, Bernhard H. Breier

**Affiliations:** 1 School of Food and Nutrition, College of Health, Massey University, Auckland 0745, New Zealand; S.N.Jayasinghe@massey.ac.nz (S.N.J.); R.Kruger@massey.ac.nz (R.K.); caoguojiao_120@hotmail.com (G.C.); staceyrivers2@gmail.com (S.R.); M.Richter@massey.ac.nz (M.R.); 2 Institute for Natural and Mathematical Sciences, College of Sciences, Massey University, Auckland 0745, New Zealand; dciwalsh@gmail.com

**Keywords:** glucose thresholds, sweet taste intensity, hedonic liking, food intake, beverage liking

## Abstract

A range of psychophysical taste measurements are used to characterize an individual’s sweet taste perception and to assess links between taste perception and dietary intake. The aims of this study were to investigate the relationship between four different psychophysical measurements of sweet taste perception, and to explore which measures of sweet taste perception relate to sweet food intake. Forty-four women aged 20–40 years were recruited for the study. Four measures of sweet taste perception (detection and recognition thresholds, and sweet taste intensity and hedonic liking of suprathreshold concentrations) were assessed using glucose as the tastant. Dietary measurements included a four-day weighed food record, a sweet food-food frequency questionnaire and a sweet beverage liking questionnaire. Glucose detection and recognition thresholds showed no correlation with suprathreshold taste measurements or any dietary intake measurement. Importantly, sweet taste intensity correlated negatively with total energy and carbohydrate (starch, total sugar, fructose, glucose) intakes, frequency of sweet food intake and sweet beverage liking. Furthermore, sweet hedonic liking correlated positively with total energy and carbohydrate (total sugar, fructose, glucose) intakes. The present study shows a clear link between sweet taste intensity and hedonic liking with sweet food liking, and total energy, carbohydrate and sugar intake.

## 1. Introduction

Taste or gustation is the sensation experienced when a substance in the mouth is recognized by taste receptors of taste buds on the tongue papillae. There are five established basic tastes; sweet, salty, sour, bitter, and umami (savory) [1]. These taste sensations influence the consumption of food (sweet, salty, umami) and may trigger the rejection of toxins (bitter, sour). Thus, taste perception influences food selection and dietary intake. Furthermore, over the past few decades there has been an increasing interest in understanding the role taste perception plays in satiety, energy balance and long-term health [2,3]. Emerging data suggests that the gustatory system may also be involved in many other important metabolic processes such as energy homeostasis and appetite regulation, thereby influencing body weight and health [4,5]. Given that sweet taste has a powerful hedonic appeal, preferences for sweet foods and beverages are important contributors of body weight and obesity development [6,7]. Obesity is a global health issue of epidemic proportion [8], and interventions to halt the epidemic have been unsuccessful [9,10]. Although the causes of obesity are complex, key drivers include over-consumption of inexpensive, highly palatable, energy-dense and nutrient-poor foods and beverages high in added sugar [11]. Global sugar intake (e.g., sucrose and high-fructose corn syrup), largely in the form of sweetened foods and beverages, has increased over the last few decades and has paralleled the increase in obesity rates [12,13].

A range of different psychophysical measurements of sweet taste is used to characterize distinct aspects of sweet taste perception [14,15]. A widely used method involves threshold testing, which determines the responsiveness of a person to sweet taste stimuli. Sweet taste thresholds can assess either the detection or the recognition threshold, measuring the lowest concentration of a sweet tastant that can be detected or recognized as sweet respectively [16]. Another commonly used psychophysical measurement of sweet taste perception is sweet taste intensity [17]. Using concentrations above the taste recognition threshold, referred to as suprathreshold concentrations, sweet taste intensity measures the sensation of intensity that is produced by the tastant at a given concentration [16]. In addition to sweet taste intensity, a hedonic measure of liking or preference is used to characterize the hedonic value of the sweet taste perception [15].

It is well known that taste has an important influence on food choices [18,19]. Furthermore, there are large variations in sweet taste perception between individuals [20], which in turn could influence their specific dietary intake. For example, individuals who have a high sweet detection or recognition threshold, and/or a lower level of perceived sweet taste intensity, may require higher quantities of a sweet tastant to be satisfied with the perception of sweet taste they are experiencing. In addition, an increased hedonic liking of sweetness at high concentrations may result in increased consumption of sweet food [21].

A number of previous studies that have investigated the relationship between sweet sensitivity (assessed using sweet taste thresholds or sweet taste intensity measurements), or hedonic liking, and food intake have reported contradicting results. While most studies reported no relationship between sweet taste sensitivity (i.e., sweet taste thresholds or sweet taste intensity measurements) and the type or the amount of sweet food consumed [17,22,23,24,25], one study showed a negative correlation between sweet (aspartame) recognition thresholds and energy intake [24]. Furthermore, a positive correlation was observed between sweet taste intensity of high intensity sweeteners stevia and sucralose, and mean total energy intake [25]. Other studies have shown that a high hedonic liking or a strong preference for sweet taste was associated with a higher habitual intake of sweet food [17], an increased consumption of sweet beverages [22], and the sugar content of preferred sugar-rich cereals [26]. It is likely that discrepancies between studies due to differences in the study participants (gender, ethnicity, age), assessment methods of sweet taste perception (psychophysical measurement, type of sweet stimuli) or dietary intake assessment methods (food record, food frequency questionnaire), generate inconsistencies about the potential biological or functional relationships. The increasing interest in the role of sweet taste perception and its influence on sweet food intake has led to a proliferation of studies using a range of sweet taste measurements including taste thresholds, intensity and hedonic liking [17,25,27]. Many of these measurements, however, have not been rigorously assessed and may limit the value of the data obtained. Furthermore, there is a dearth of data that systematically compare the outcomes of different sweet taste perception measurements to each other and how they might relate to dietary intake and eating behavior [14,25,28].

The present study had the overall goal to increase our understanding of the biological and functional links between sweet taste perception and sweet food intake. The specific aims were; firstly, to evaluate and compare four widely used psychophysical measurements that characterize sweet taste perception; and secondly, to explore whether any of these assessments of sweet taste perception may relate to sweet food intake. A better understanding of the different assessment methods of sweet taste perception, and a thorough evaluation of their relationships to each other and with sweet food intake will provide new insights into the role that sweet taste perception plays in habitual sweet food liking and intake, and how it may influence long-term health.

## 2. Materials and Methods

### 2.1. Study Participants

The study was conducted in accordance with the Declaration of Helsinki, and the protocol was reviewed and approved by the Massey University Human Ethics Committee. All participants provided written informed consent prior to participating in the study. Forty-four healthy New Zealand European women, aged 20–40 years, were recruited from the wider Auckland area. Participants were excluded if pregnant or breastfeeding, currently on any diet that excludes food groups (e.g., vegan), smoking or in the process of quitting, diagnosed with any metabolic illness, have conditions that could alter gustatory functions (e.g., chemotherapy), have any form of oral or nasal disease, currently taking medication that may influence taste perception or saliva production, or have taken antibiotics in the past three months [29].

### 2.2. Study Procedure

Participants visited the Human Nutrition Research Unit and sensory research facilities at Massey University Auckland on four separate sessions, at least a day apart and within a month (mean ± SD: 21 ± 11 days). Four psychophysical measurements of sweet taste were determined at each session using glucose as the sweet tastant. These included; detection threshold, recognition threshold, sweet taste intensity, and hedonic liking. Glucose was used as the sweet tastant as it is a simple sugar, has clearly defined metabolic links with glucose metabolism [30], and the potential involvement of taste cell-expressed glucose sensors and ATP-gated K+ channels as mediators of a type 1 taste receptor-independent sweet taste of sugars [31]. In addition, participants completed a four-day weighed food record, sweet food-food frequency questionnaire (SF-FFQ), and a sweet beverage liking questionnaire pertaining to food intake and sweet beverage liking. All four sessions were standardized by conducting the taste evaluation between 7 and 10 a.m. after an overnight fast [32] to ensure sweet taste perception measurements were not influenced by hormonal fluctuations and different levels of hunger. Participants were asked to abstain from brushing their teeth one hour prior to testing, and were tested during any stage of their menstrual cycle [32,33]. At the first session participants completed a health and demographic questionnaire, and their height (stadiometer), weight and body fat percentage (Bioelectrical Impedance Assessment (BIA) InBody 230, Biospace, Cerritos, CA, USA) were measured.

### 2.3. Sweet Taste Perception Measurements

At each of the four sessions, participants first completed the psychophysical measurements of glucose detection and recognition thresholds, followed by rating the sweet taste intensity and hedonic liking of four suprathreshold glucose concentrations. Sweet solutions were prepared on the day of testing by dissolving glucose (dextrose monohydrate, Qinhuangdao Lihua Starch Co. Ltd., Qinhuangdao, China) in distilled water. Sensory testing was conducted in individual sensory booths at room temperature (20 °C). Participants tasted the samples using the whole mouth testing (sip and spit) method [24], and did not wear nose clips during sensory testing.

#### 2.3.1. Glucose Detection and Recognition Thresholds

Eight glucose concentrations, ranging between 15–180 mM (15, 30, 45, 60, 90, 120, 150, 180 mM) were prepared to determine detection and recognition thresholds. This concentration range covered the published glucose detection and recognition thresholds [32,34], and allowed for inter-individual threshold differences. Participants received the samples at each concentration as a 3-alternative forced-choice ascending series [35]. Starting from the lowest glucose concentration, three samples (10 mL each), one containing the glucose sample and two background samples (distilled water) were presented at each concentration. Participants were asked to take the whole 10 mL of sample in to their mouth, swirl the solution around for 3 s and expectorate. Using the forced-choice method, participants selected the sample with the sweet taste. Participants evaluated each glucose concentration three times at the same concentration level before moving on to the next higher concentration, completing 24 trials in total at each of the four sessions. All samples were given a three-digit number, and the position of the sweet sample was randomly allocated. Participants were asked to rinse their mouth with distilled water between each concentration step.

#### 2.3.2. Sweet Taste Intensity and Hedonic Liking of Suprathreshold Glucose Concentrations

Each participant rated the sweet taste intensity and hedonic liking of 125, 250, 500 and 1000 mM glucose samples (10 mL each) presented in a random order. Sweet taste intensity was rated on a 100 mm general Labeled Magnitude Scale (gLMS) ranging from no sensation (0 mm) to strongest imaginable sensation (100 mm) with verbal descriptors assigned to different levels of intensities [36]. Hedonic liking was rated on a hedonic gLMS ranging from dislike extremely (−100 mm) to like extremely (+100 mm) with neutral (0 mm) in the middle [37]. Participants were instructed and trained on how to use both gLMS scales according to the protocol outlined by Green et al. (1996) [36], and encouraged to mark anywhere on the scale in accordance with the level of taste perception they experienced. For example, if the sweet taste intensity of a sample was perceived between moderate and strong, they were asked to mark closer to the verbal descriptor that more closely represented the sweet sensation perceived.

### 2.4. Dietary Measurements

#### 2.4.1. Food Intake

Each participant completed a four-day weighed food record to assess their food intake [38,39]. Participants were given verbal instructions and shown a food record video on how to maintain a weighed food record. They were instructed to record what was consumed, time of consumption, quantity consumed and how the food was prepared. All participants were given a food record booklet, electronic scales and a food portion booklet containing portion sizes (used when scales cannot be used e.g., dining out). Participants recorded their dietary intakes on four days consisting of one weekend day and a maximum of 2 consecutive days. On each of the testing sessions, the previous day’s food record was checked for accuracy and completeness.

#### 2.4.2. Frequency of Sweet Food and Beverage Intakes

The SF-FFQ was specifically developed to assess the frequency of sweet food and sweetened beverage consumption over the previous month [25]. The main purpose of the SF-FFQ was to assess the habitual intake (in terms of frequency) of sweet tasting foods rather than quantifying their intake. The sweet foods included in the questionnaire were based on the 1997 and 2008/2009 New Zealand national nutrition surveys [40,41]. Participants indicated their frequency of intakes of 69 sweet tasting foods and beverages (both natural sweet tasting foods and processed sweetened foods) using the following frequencies: never, less than once a month, 2–3 times per month, once per week, 2–4 times per week, 4–6 times per week, once a day, and twice or more a day [42,43].

#### 2.4.3. Sweet Beverage Liking

A sweet beverage liking questionnaire was developed to measure hedonic preferences of 16 sweet tasting beverages [21]. The sweet beverages included in the questionnaire were based on data from the 1997 and 2008/2009 national nutrition surveys, where beverages were listed as major sources of sugar intake of New Zealanders [40,41]. Participants rated the liking of the sweet beverages on a 100 mm visual analogue scale anchored between strong dislike and strong like. Pictures and examples of the sweet beverages were available to assist participants in completing the task.

### 2.5. Data Handling and Statistical Methods

#### 2.5.1. Sweet Taste Perception Measurements

Glucose detection and recognition thresholds were determined from the sweet taste threshold detection curves produced for each participant by graphing the probability of detection levels against the glucose concentrations. A best-fit curve for each participant was then fitted using a logistic regression model. This method was a modified graphical approach similar to that used by Lawless 2010 [44]. As the response variable (success or failure to choose the correct sample with the sweet taste) is binomial, a logistic regression model was used to predict the probability that each individual would successfully detect the sweet sample at each concentration. The model used a common intercept for all participants, but the slopes were estimated separately for each participant based on their results from the four sessions. The values of the fitted parameters (slopes and intercepts) estimated for each participant calculated for a range of concentrations was used to create sweet taste detection curves. Using the sweet taste detection curves, glucose concentrations (x-axis) corresponding to different probability of detection levels (y-axis) were interpolated to determine individual glucose thresholds. Interpolation at a probability of 0.8 was chosen as the detection threshold to represent threshold values obtained if a predetermined stopping rule of three consecutive correct identifications at one concentration was used [45]. Interpolation at 0.99 probability of detection was chosen to represent recognition threshold.

Sweet taste intensity ratings were measured from 0 to 100 mm and hedonic liking ratings from −100 mm (dislike extremely) to +100 mm (like extremely). The average detection and recognition thresholds, sweet taste intensity and hedonic liking measurements over the four repeated sessions were used to explore relationships between sweet taste measurements, and between sweet taste and dietary measurements.

#### 2.5.2. Dietary Measurements

All weighed food record data were entered in to FoodWorks 7 (FoodWorks Professional 2013, Xyris Software), using the New Zealand Food Composite Database to determine total energy and macronutrient intakes. Carbohydrate intakes were differentiated into starch (polysaccharides) and total sugars (free and added mono- and di-saccharides). Participants whose daily energy intakes were outside 1000–5000 kcal (i.e., 4200–21,000 kJ) were excluded from the analysis [46,47]. A total of forty-three participants completed food records of which two participant’s food records were excluded from the analysis due to under-reporting (<1000 kcal).

The SF-FFQ intakes of the 69 food items were converted to daily frequency equivalents (DFE) calculated by allocating proportional values to the original frequency categories with reference to a base value of 1.0, equivalent to once a day [42,43]. The scores were calculated as follows: DFE score of 2—twice a day or more; 1—once per day; 0.71—4–6 times per week; 0.3—2–4 times per week; 0.14—once per week; 0.08—2–3 times per month; 0.03—Less than once a month; 0—never. The 69 food items were categorized into eight main sweet food categories; fruit, vegetables, dairy, spreads/sweeteners, cereals, baking/sweets, desserts, and beverages, and a median DFE score for each category was determined. Finally, a single DFE score for the frequency of total sweet food intake was also calculated.

The beverage liking questionnaire was assessed by measuring a liking score (in mm) from zero for each sweet beverage ranging from strong dislike (−50 mm) to strong like (+50 mm).

#### 2.5.3. Statistical Analysis

All measurements related to sweet taste threshold detection curves were conducted using the R statistical program version 3.2.5. All other data analysis was conducted using SPSS software version 23 (IBM Corporation, New York, NY, USA). All continuous variables were tested for normality using Kolmogorov-Smirnov test together with analyzing histograms, normal Q-Q plots and boxplots. Non-normal data were log transformed and re-tested for normality. Parametric data are presented as mean ± standard deviation (SD), and non-parametric data as median [25,75 percentiles]. Log transformed data are reported as mean [95% confidence intervals] (CI). Intraclass correlation coefficient (ICC) (two-way random effects model, absolute agreement, average measures) was used to assess the between-session correlation of thresholds, sweet taste intensity, and hedonic liking over the four repeated sessions. An ICC value >0.7 was considered good correlation, while a ICC <0.7 was considered moderate to low correlation [48]. Repeated measures design was used to test differences in sweet taste intensity and hedonic liking ratings between glucose concentrations. Relationships between two continuous variables were tested using the Pearson’s correlation coefficient for parametric data and Spearman’s correlation coefficient for non-parametric data. Correlation coefficient were used to determine the strength of the relationship by the criteria; ±0.1 = weak, ±0.3 = moderate and ±0.7 = strong [49]. A *p* < 0.05 was considered statistically significant. All statistical tests were 2-tailed.

## 3. Results

### 3.1. Participant Characteristics

Participants had a mean (±SD) age of 28 ± 6 years and a mean [95% CI] body weight of 66.6 [63.8, 69.5] kg. The mean [95% CI] BMI and body fat were 24.0 [23.0, 25.1] kg/m^2^ and 30.2 [28.2, 32.2] % respectively.

### 3.2. Sweet Taste Threshold Detection Curves

**Figure 1:**
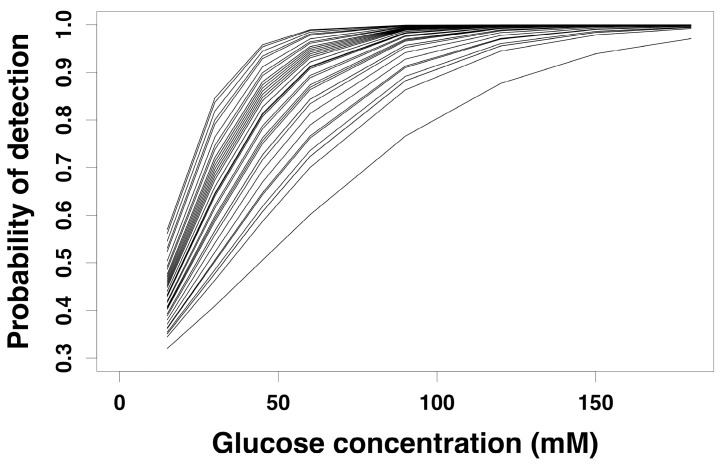
Sweet taste threshold detection curves of all participants. Each line of this figure represents the best-fit curve of each participant generated by a binomial regression model with a common intercept and separate slopes. The figure was generated from the average threshold data of the four repeated sessions. *n* = 44.

Figure 1 shows the sweet taste threshold detection curves of each participant describing the sweet taste detection at different glucose concentrations. The detection curves were produced by calculating the probability of sweet taste detection against the glucose concentrations using data from all four sessions. A binomial logistic regression model was used to determine the best-fit curve for each participant and interpolate glucose detection and recognition thresholds.

As expected, all participants showed increased sweet taste detection with increasing glucose concentrations, which was observed across all four sessions. However, the rate of sweet detection, identified as the concentration at which each participant was able to consistently select the sweet sample, differed markedly between participants. The specific rate of detection for each participant was used as a marker of sweet sensitivity to interpolate glucose thresholds. Each participant’s detection and recognition thresholds were determined by interpolating the glucose concentrations corresponding to different probability of detection levels using their own detection curve. Interpolation at a probability of detection of 0.8 was chosen as the detection threshold, and a probability of detection of 0.99 was chosen as recognition threshold.

### 3.3. Glucose Detection and Recognition Thresholds

The group median detection and recognition thresholds at each of the four sessions are reported in Table 1. The repeatability of glucose thresholds over the four sessions, tested using intraclass correlation coefficient, indicated strong between-session correlations (Table 1).

### 3.4. Sweet Taste Intensity and Hedonic Liking at Suprathreshold Concentrations

The group mean sweet taste intensity and hedonic liking ratings of the four glucose concentrations at each of the four sessions are reported in Table 2. As expected, at each session, participants perceived the sweet taste as more intense with increasing glucose concentrations. Overall, across all sessions there was no significant difference in hedonic liking between 125 mM and 250 mM glucose, but a significant decrease in hedonic liking was observed at the higher glucose concentrations, 500 mM and 1000 mM glucose (Table 2). The between-session repeatability of sweet taste intensity and hedonic liking, assessed by intraclass correlation coefficient, increased as the glucose concentrations increased, with sweet taste intensity and hedonic liking of 1000 mM glucose showing the highest between-session ICC (Table 2).

**Figure 2:**
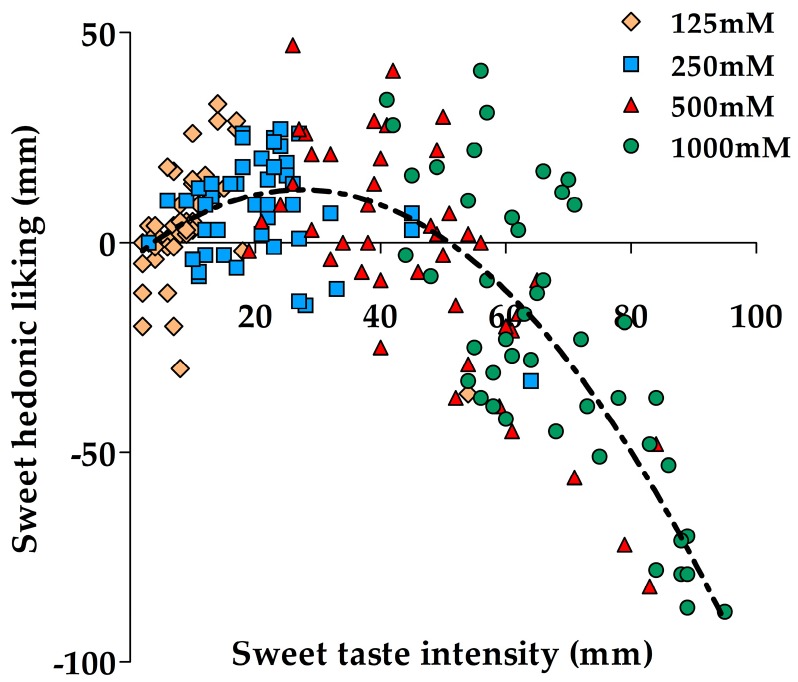
Scatterplot of the relationship between sweet taste intensity and hedonic liking. The scatterplot was generated using the average sweet taste intensity and hedonic liking ratings across the four repeated sessions. *n* = 44.

The suprathreshold sweet taste intensity and hedonic liking ratings at any glucose concentration did not correlate with glucose detection or recognition thresholds. However, the relationship between sweet taste intensity and hedonic liking showed a concentration-dependent bi-phasic relationship. Figure 2 shows the scatterplot of the relationship between sweet taste intensity and hedonic liking across all four glucose concentrations generated using the average ratings across the four repeated sessions. Importantly, the relationship between sweet taste intensity and hedonic liking changed with increasing glucose concentrations. A positive relationship was present at the lowest glucose concentration 125 mM (*r_s_* = 0.52, *p* < 0.001), followed by no relationship at 250 mM, and shifting to a clear negative relationship at the two highest concentrations, 500 mM (*r* = −0.75, *p* < 0.001) and at 1000 mM (*r* = −0.76, *p* < 0.001).

### 3.5. Food Intake

The study population’s mean daily intakes of total energy, macronutrients and total sugars, obtained using four-day weighed food records are reported in Table 3. Intakes of macronutrients and sugars are expressed as absolute intakes in grams and as a percentage of total energy intake. The mean energy intake of the group (7698 kJ) was slightly lower than the estimated energy requirement (8000–8400 kJ) for women of similar age and weight [50]. With reference to the acceptable macronutrient distribution range (AMDR) for carbohydrate (45–65%), protein (15–25%) and fat (20–35%) [50], the average intakes of this group were slightly lower for carbohydrate intake (42%) and higher for fat intake (37%). Furthermore, sucrose was the predominant sugar subgroup contributing to 8% of total energy intake.

### 3.6. Frequency of Sweet Food Intake

Table 4 shows the frequency of daily intakes of the eight sweet food categories assessed using the SF-FFQ. On average, each participant consumed a combination of naturally sweet foods and foods with added sugars or sweeteners seven times a day. The three most frequently consumed food categories were fruit, baking/sweets and sweet beverages, and the least consumed sweet categories were sweet vegetables and desserts.

### 3.7. Sweet Beverage Liking

The mean liking scores for all 16 sweet beverages are reported in Table 5. The three most liked sweet beverages were fruit smoothies, cocktails and dessert wine/ciders. The least liked drinks were fruit drinks, cordials and energy drinks.

### 3.8. Relationship between Sweet Taste Perception and Dietary Measurements

#### 3.8.1. Sweet Taste Perception and Food Intake

There was no correlation between sweet taste detection and recognition thresholds, and total energy intake and intakes of macronutrients and sugars in grams or expressed as a percentage of total energy (*p* > 0.05). The correlation between sweet taste intensity and energy, macronutrients and sugar intakes are reported in Table 6. There was a clear dose-dependent negative correlation between sweet intensity perceived at 250 mM, 500 mM and 1000 mM glucose, and total energy, and carbohydrate, starch, total sugar and glucose intakes in grams. However, sweet taste intensity at any glucose concentration was not correlated with macronutrients and sugars intakes when expressed as a percentage of total energy intake (*p* > 0.05).

The correlations between sweet hedonic liking and intake of energy, macronutrients and sugars are reported in Table 7. A positive correlation was observed between hedonic liking of 500 mM and 1000 mM glucose, and total energy, carbohydrate, total sugar, fructose, glucose and maltose intakes in grams. However, when expressed as a percentage of total energy, only with intakes of total sugar and maltose were positively correlated with sweet taste intensity of 1000 mM.

#### 3.8.2. Sweet Taste Perception and Frequency of Sweet Food Intake

**Figure 3:**
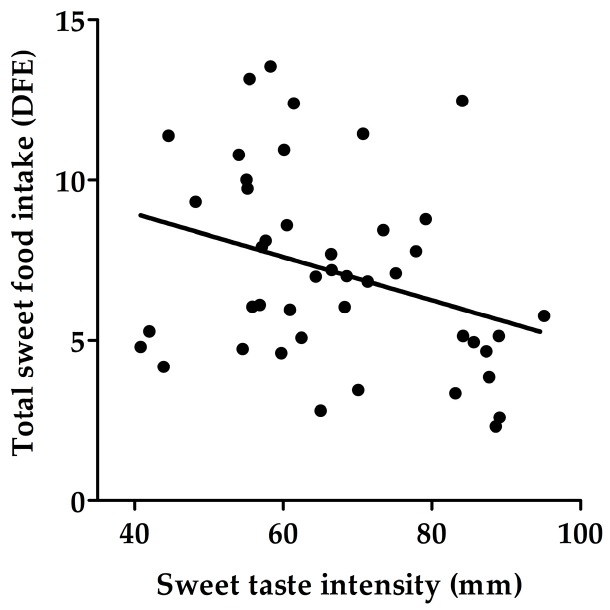
Relationship between sweet taste intensity of 1000 mM glucose and frequency of total sweet food intake. DFE: daily frequency equivalent. *r* = −0.33, *p* = 0.03. *n* = 44.

No significant correlations were found between detection threshold, recognition threshold or hedonic liking, and the frequency of intake of any sweet food category. However, there was a negative correlation between the frequency of baking/sweets intake and sweet taste intensity perceived at 125 mM (*r*_s_ = −0.44, *p* = 0.003), 250 mM (*r*_s_ = −0.43, *p* = 0.003), 500 mM (*r*_s_ = −0.37, *p* = 0.01) and 1000 mM (*r*_s_ = −0.31, *p* = 0.04) glucose. Furthermore, perceived sweet taste intensity of 500 mM and 1000 mM glucose was negatively associated with the frequency of total sweet food intake (*r* = −0.33, *p* = 0.03, Figure 3).

#### 3.8.3. Sweet Taste Perception and Sweet Beverage Liking

**Figure 4:**
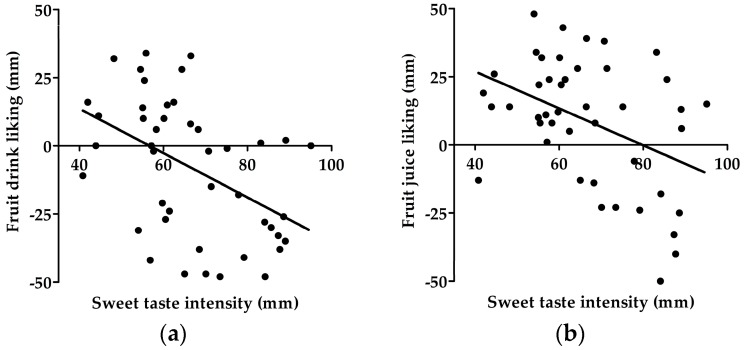
Relationship between sweet taste intensity perceived at 1000 mM glucose and sweet beverage liking: (**a**) Fruit drink (*r* = −0.42, *p* = 0.005); (**b**) Fruit juice (*r* = −0.47, *p* = 0.001). *n* = 44.

Correlation analysis showed that liking of fruit drink was negatively correlated with perceived sweet taste intensity of 500 mM (*r* = −0.32, *p* = 0.03), and 1000 mM (*r* = −0.42, *p* = 0.005, Figure 4a), and positively correlated with sweet hedonic liking of 500 mM (*r* = 0.35, *p* = 0.02), and 1000 mM glucose (*r* = 0.34, *p* = 0.02). Furthermore, liking of fruit juice was negatively correlated with the perceived intensity of 500 mM and 1000 mM glucose (*r* = −0.47, *p* = 0.001, Figure 4b).

## 4. Discussion

The present study evaluated the relationship between four widely used assessment methods of sweet taste perception and investigated the link between these sweet taste measurements and sweet food liking and intake. This paper had four main findings. Firstly, detection and recognition thresholds showed no correlations with perceived sweet taste intensity or hedonic liking. Secondly, a dose-dependent change in the relationship between sweet taste intensity and hedonic liking of suprathreshold concentrations indicated that a sweet tastant is liked at a lower concentration and disliked at higher concentrations in a dose-dependent manner. Thirdly, although individual participants showed distinct patterns of sweet detection, glucose detection and recognition thresholds were not correlated with intakes of energy, macronutrients and sugars, frequency of sweet food intake or sweet beverage liking. Lastly, total energy intake, and absolute intake of carbohydrate (i.e., starch, total sugar, fructose and glucose) correlated negatively with sweet taste intensity and positively with hedonic liking of suprathreshold glucose concentrations in a dose-dependent manner. To the best of our knowledge this is the first study to report robust relationships between sweet taste intensity and hedonic liking of suprathreshold glucose concentrations, and food intake and sweet beverage liking. These findings have implications for eating behavior and long-term health outcomes, as sensory properties of foods and beverages clearly influence preferences, and the type and amount of food consumed.

### 4.1. Inter-Individual Variations in Sweet Taste Perception Measurements

In the present study, detection and recognition thresholds were interpolated from sweet taste threshold detection curves produced for each participant. This is the first study that determines glucose thresholds using the sweet taste threshold detection curve method. Although the detection of sweet taste of all participants increased with increasing glucose concentration, each participant had a distinct sweet detection curve, with a distinct rate of sweet taste detection. This indicates clear inter-individual variations in sweet sensitivity. The median glucose detection (41.3 mM) and recognition (91.0 mM) thresholds in the present study were similar to previously published mean detection and recognition thresholds of 54 mM [34] and 95.3 mM [32] respectively, but they were higher compared to another study with the reported mean glucose detection and recognition thresholds of 17.2 mM and 35.2 mM respectively [25].

It is well known that methodological details associated with threshold testing contribute to the inherent variability of the data between different laboratories making it difficult to compare threshold values between studies. The stopping rule, which determines the point of termination of the taste testing, also influences the allocation of threshold values [51]. One of the strengths of the present study is that glucose thresholds obtained from the sweet taste threshold detection curves used no predetermined stopping rule and therefore did not rely on a stopping rule purely determined by chance [44]. As seen from the data of the present study, the detection of sweet taste does not change from no-detection (probability zero) to detection (probability one), but rather describes a gradual increase in detection with increasing concentrations. Therefore, by using each participant’s dose-response curve, more accurate sweet taste thresholds can be determined. Furthermore, the intraclass correlation coefficient showed good between-session repeatability for detection (ICC = 0.64) and recognition (ICC = 0.67) thresholds, similar to the sucrose detection thresholds (ICC = 0.66) reported in a previous study [48]. This shows that glucose thresholds were consistent and reproducible over repeated sessions in this study.

In the present study, perceived sweet taste intensity increased with increasing glucose concentrations, suggesting that the glucose concentrations were able to clearly evoke different levels of sweet taste intensities. Furthermore, the hedonic liking ratings indicated that participant’s liking of sweet taste was concentration-dependent. The current study found that the between-session correlations for both sweet taste intensity and hedonic liking increased with increasing glucose concentrations, similar to a previous study where higher repeatability of perceived pleasantness ratings were observed for solutions with higher sugar levels [52]. This suggests that an individual’s ability to consistently perceive the intensity or hedonic liking of a sweet solution is greater when the strength of the sweet signal is stronger (i.e., higher concentrations), possibly due to the increased saturation of the receptor-ligand interactions of sweet taste receptors [53].

The inter-individual phenotypic variations in sweet taste thresholds, sweet intensity and hedonic liking observed in the present study could possibly be explained by the genetic variation of the taste receptor type 1 and 3 (T1R2 and T1R3) subunits of the G-protein coupled receptor responsible for sweet taste [54]. In particular, single nucleotide polymorphisms of the T1R2 and T1R3 receptors have been associated with variations in sweet taste perception and consumption of sweet foods [55,56]. Although genetic variation was not investigated in the present study, this emerging field of research could provide further understanding of the inter-individual differences in sweet taste perception.

### 4.2. Relationship between Sweet Taste Perception Measurements

The present study found no correlation between the glucose thresholds, and sweet taste intensity or hedonic liking of any suprathreshold glucose concentration similar to previous studies using sweet, sour and salty, and caffeine tastants [14,28,57]. These results suggest that a person with a low detection or recognition threshold for a sweet tastant may not necessarily experience a greater sweet sensation or like sweet taste at low concentrations. The lack of relationship between thresholds and suprathreshold measurements suggest that each psychophysical measurement characterizes a specific feature of sweet taste. As already discussed by Webb et al. (2015), one sweet taste measure alone is not a convincing marker of overall sweet taste perception. Therefore, a combination of sweet taste measurements may provide a better understanding of the sense of taste, and enhance the inquiry about relationships between taste perception and food intake [14]. One of the important findings of the present study describes the change in the relationship between sweet taste intensity and hedonic liking with increasing concentrations of sweet tastant, starting with a positive relationship at the lowest glucose concentration, and moving to a negative relationship at the two highest glucose concentrations. This relationship between sweet taste intensity and liking at the lowest (125 mM) glucose concentration shows that participants who perceived this concentration as sweet, liked the level of sweetness more than those who experienced the same concentration as less sweet. Importantly, participants who perceived the two highest glucose concentrations as more sweet disliked the sweetness more than participants who perceived the solutions as less sweet. This change in the relationship between sweet taste intensity and liking suggests that the intensity measurements at suprathreshold concentrations relate more strongly to the hedonic experience. Furthermore, the finding that participants generally disliked the two highest concentrations has implications for our food environment, because these levels of sugars or other sweeteners are commonly found in sweet beverages. Our study suggests that there is ample scope to reduce the sugar content in sugar-sweetened beverages but still maintain hedonic liking.

### 4.3. Characterising Food Intakes of the Study Participants

The four-day weighed food record data indicated that the study participants on average consumed relatively low levels of carbohydrate, but high levels of fat, and moderate levels of protein. The mean energy intake of the group (7698 kJ) was slightly lower than the estimated energy requirement (8000–8400 kJ) for women of similar age and weight [50]. Furthermore, total sugar (88.9 g) and sucrose (38.8 g) intakes were generally lower than the mean total sugar (97–121 g) and sucrose (43.6–61.6 g) intakes of a similar population in New Zealand [41]. According to the SF-FFQ data from the present study, the most frequently consumed sweet food categories were fruit, baking/sweets and sweet beverages, and as indicated by the sweet beverage liking questionnaire, fruit smoothie was the most liked sweet beverage. Together these data indicate that the participants of the present study were consuming a relatively healthy diet with moderate intakes of fructose and glucose and relatively low intake of sucrose.

### 4.4. Suprathreshold Taste Measurements and Food Intake

To the best of our knowledge this is the first study to report robust significant relationships between sweet taste intensity and hedonic liking at suprathreshold concentrations of a sweet tastant, and food intake. Previous studies have failed to find associations between sweet taste and diet parameters [15,24,25,28]. One of the strengths of the food intake data from the present study is that it was derived from in-depth weighed food records, which is considered the ‘gold standard’ method for quantifying nutrient intake [39]. The dose-dependent negative correlation between sweet taste intensity, and total energy intake and absolute intakes of carbohydrate (as well as starch, total sugar, fructose and glucose) in the present study suggests that participants who perceived glucose solutions as more sweet have lower energy, carbohydrate and sugar intakes in comparison with participants who perceived the glucose solutions as less sweet. This is supported by findings of a recent study that showed that reduced perceived intensity correlated with increased desire for higher calorie taste stimuli [58].

The significant positive correlation between hedonic liking of 500 mM and 1000 mM glucose, and total energy and absolute intakes of carbohydrate (as well as total sugar, fructose, glucose) suggests that participants who have a higher hedonic liking for sweet taste consumed more energy, carbohydrates and especially more sugars. This positive association supports previous research where a positive relationship was found between hedonic liking of sweet taste, and liking ratings of sweet desserts and sugar in tea [15], frequency of sweet food consumption, intake of refined and total sugars [17], and sugar content of favorite cereals [26].

Interestingly, this study found no relationship between sweet taste intensity and hedonic liking, and fat or protein intake, illustrating the clear link between sweet taste and intake of sweet tasting food. Furthermore, the lack of correlation between suprathreshold sweet measurements, and sucrose intake could be attributed to the generally lower intake of sucrose in this group of women. We also observed no correlations between sweet taste intensity and hedonic liking, and macronutrient intakes expressed as a percentage of total energy. Due to the significant relationship with total energy, it is not surprising that the relationship between sweet taste intensity and hedonic liking, and macronutrient intake were not significant when adjusted for total energy intake. A similar finding was reported by Stewart et al. (2011), where a significant difference in absolute intakes of fat and saturated fat between oleic acid hyper- and hypo-sensitive groups resulted in a loss of significant differences after adjusting fat intakes for total energy [59]. The same study suggested that because fatty acids interact directly with the taste receptors, the absolute intake of fat was more biologically relevant than the energy adjusted macronutrient intakes when assessing relationships between taste and food intake [59]. Nevertheless, the findings of our study illustrate an important biological relationship between how sweet taste is perceived and liked, and the intake of energy and macronutrients even within this group of young women with relatively healthy diets.

### 4.5. Suprathreshold Taste Measurements, Frequency of Sweet Food Intake and Sweet Beverage Liking

The data from the SF-FFQ and sweet beverage liking questionnaires showed a clear negative correlation between sweet taste intensity of 500 mM and 1000 mM glucose, and frequency of total sweet food intake (as well as the frequency of baking/sweets intake) and liking of fruit juice and fruit drink. This suggest that participants who perceived the highest glucose concentrations as more sweet are more sensitive to sweet taste and therefore had a lower frequency of sweet food intake and lower preferences for sweetened beverages compared to those who perceived the solution as less sweet.

The sweet beverage-liking questionnaire also showed that fruit drink liking was correlated positively with the hedonic liking of 500 mM and 1000 mM glucose, suggesting that participants who have a higher hedonic liking for sweet taste have increased liking for this sweet beverage. It is not surprising to find no other significant relationships between the hedonic liking of the glucose solutions and other sweet beverages, as the sensory experience of taste testing in a laboratory setting is different, and does not perfectly emulate sensory experiences of real-world beverages. For example, the sweet tastants in our study were present at room temperature while sweet beverages in the real world are usually served cold to increase its palatability and sensory properties [60]. We believe this contributed to the lack of relationship between hedonic liking of glucose solutions and liking of sweetened beverages.

### 4.6. Glucose Thresholds, Food Intake and Sweet Beverage Liking

Although sweet taste threshold detection curves showed clear inter-individual variations in sweet taste detection, no correlations were found between glucose detection and recognition thresholds, and any dietary measurement. This finding is consistent with previous studies where no relationships were found between sweet taste thresholds and food intake [24,25,28]. It has previously been discussed that taste thresholds are poor predictors of taste experienced with real world foods, because taste thresholds measure the lowest concentration of a tastant detected or recognized [57,61]. This phenomenon may also explain the lack of an association between sweet taste thresholds and dietary measurements observed in the present study.

### 4.7. Summary

The present study shows significant relationships between dietary measurements and both sweet intensity and hedonic liking of suprathreshold concentrations, as previous studies have only found links between dietary measurements and sweet intensity [25] or between dietary measurements and hedonic liking [17,22,26]. The correlations found in this study can only establish a link between sweet taste intensity with increased sweet food intake, and cannot establish the direction of this relationship. It is possible that either a habitually high sweet food intake contributes to lower perception of sweet taste intensity, or, a lower sweet intensity perception may lead to increased habitual sweet food intake. However, this significant relationship is consistent with a recent study reporting sweet intensity to be the most appropriate measure to assess links between sweet taste and food intake [25]. Furthermore, a recent dietary intervention study showed that participants rated pudding samples as sweeter following a three-month diet of reduced sugar intake, indicating sweet taste intensity to be an important factor associated with habitual food intake [62]. It is tempting to speculate that this relationship may be modifiable by dietary changes.

The current food environment exposes individuals to strong cues that favor energy availability and a positive energy balance, which can lead to obesity and other metabolic disorders (e.g., type 2 diabetes) [6,7]. Commonly cited causes of obesity include major changes in our food environment which have led to over-consumption of highly palatable energy-dense, nutrient-poor and inexpensive foods with a noticeable increase in sugar intake over the past 30 years [13,63]. Our data suggest that sweet taste intensity and hedonic perceptions of suprathreshold concentrations may play a biological role in dietary intake (energy and carbohydrates), frequency of sweet food consumption and sweet beverage liking, thereby influencing body weight and long-term health. The nature (cause or effect) of this relationship requires further investigation.

The strengths of the present study include a thorough comparison of four commonly used psychophysical measurements of sweet taste perception, and an investigation of the relationship between all four sweet taste measurements with a range of parameters of sweet food intake. Furthermore, the range of dietary assessments investigated actual food intakes (food record), habitual intakes of sweet foods (SF-FFQ), and liking of sweet beverages, capturing different aspects of food intake and liking. The present study has several limitations that require further study. Firstly, participants of the study were a small sample of New Zealand European women of similar age (young) and BMI (normal range). Therefore, the findings of this study cannot be generalized to other ethnicities, ages or BMI groups. Secondly, the study design was cross-sectional and the findings show only relationships and no causations can be ascertained. Thirdly, the participants of this study consumed a relatively healthy diet with generally low carbohydrate intakes and did not consume excessive amounts of sweet food. Therefore, the sample of dietary data in the present study may represent a relatively healthy spectrum of normal intakes. Lastly, the study used glucose as the sweet tastant and findings may differ if other sweet tastants are used.

## 5. Conclusions

Our study has several important findings. The changing relationship between sweet taste intensity and liking with increasing glucose concentrations illustrates a clear relationship that is dependent on an individual’s perception of sweet taste intensity. Furthermore, individuals who perceive glucose solutions as more sweet have lower intakes of energy and carbohydrate (starch, total sugars, fructose, glucose), as well as a lower frequency of sweet food intake and lower liking for sweet beverages compared to those who perceive the glucose solutions as less sweet. This notion is in agreement with a positive relationship between sweet hedonic liking and total energy and carbohydrate (total sugar, fructose, glucose) intakes, confirming that individuals who like the sweetness of the high glucose concentrations have higher habitual intakes of energy and sugars.

The present study shows a clear link between sweet taste intensity and hedonic liking, and dietary measurements in a group of young healthy women with normal BMI and relatively healthy food intakes. Stronger correlations between sweet taste and dietary measurements may exist in groups of women with higher variations of sweet food intake, especially if the sweet food intake contributes to an excess energy intake. Further research is needed to determine whether the relationships between sweet taste perception and food intake can be confirmed in other populations such as groups with different BMIs or people with healthy and unhealthy eating habits. Future studies should also employ objective measures of food intake in addition to the subjective measures used in our study. Furthermore, it is important to understand whether dietary interventions of reduced sweet food intake can change the perception of sweet taste intensity and liking. A better understanding of the relationship between sweet taste perception and dietary intake and eating behaviors will provide new insights into taste-related eating habits that may influence long-term health.

## Figures and Tables

**Table 1:** Glucose detection and recognition thresholds at the four different sessions.

	Detection Threshold (mM)	Recognition Threshold (mM)
Session 1	40.1 [30.5, 62.3]	84.6 [64.4, 131.4]
Session 2	40.6 [37.7, 47.8]	90.2 [83.8, 106.4]
Session 3	41.0 [36.7, 49.3]	93.7 [83.8, 112.7]
Session 4	40.8 [36.9, 45.9]	90.1 [81.5, 101.5]
Median of all sessions	41.3 [38.7, 51.1]	91.0 [85.5, 111.6]
ICC average measures ^a^	0.64 [0.43, 0.79]	0.67 [0.47, 0.80]

ICC: intraclass correlation coefficient. All threshold data are interpolated from the sweet taste threshold detection curves and reported as median [25, 75 percentiles]. ICC values reported as mean [95% CI]. ^a^ *p* < 0.001 for all between-session ICC measurements. *n* = 44.

**Table 2:** Sweet taste intensity and hedonic liking ratings at the four different sessions.

	**Sweet Taste Intensity (mm)**			
	125 mM	250 mM	500 mM	1000 mM
Session 1 rating	10 ± 9	23 ± 15	48 ± 19	67 ± 17
Session 2 rating	7 ± 6	19 ± 11	40 ± 16	64 ± 18
Session 3 rating	8 ± 6	21 ± 15	47 ± 19	66 ± 16
Session 4 rating	8 ± 10	19 ± 14	45 ± 24	70 ± 18
Average of all sessions	9 ± 8	21 ± 11	46 ± 16	67 ± 14
ICC average measures ^a^	0.65 [0.44, 0.80]	0.61 [0.38, 0.78]	0.81 [0.70, 0.90]	0.84 [0.74, 0.91]
	**Sweet Hedonic Liking (mm)**			
	125 mM	250 mM	500 mM	1000 mM
Session 1 rating	4 ± 19	7 ± 19	−5 ± 28	−20 ± 34
Session 2 rating	4 ± 18	7 ± 15	−1 ± 30	−22 ± 38
Session 3 rating	4 ± 21	9 ± 20	−2 ± 39	−17 ± 45
Session 4 rating	4 ± 17	7 ± 19	−7 ± 35	−32± 41
Average of all sessions	4 ± 14	8 ± 13	−4 ± 28	−23 ± 35
ICC average measures ^a^	0.78 [0.65, 0.87]	0.67 [0.48, 0.81]	0.88 [0.81, 0.93]	0.90 [0.84, 0.94]

ICC: intraclass correlation coefficient. Sweet taste intensity and hedonic liking reported as mean ± SD. ICC values reported as mean [95% CI]. ^a^ *p* < 0.001 for all between-session ICC measurements. *n* = 43.

**Table 3:** Daily intakes of total energy, macronutrients and sugars.

Energy/Nutrients	Intake
Total energy (kJ)	7698.0 ± 1716.9
Protein (g)	78.9 ± 18.4
Protein (%) ^a^	17.8 ± 4.0
Fat (g)	77.4 ± 22.1
Fat (%) ^a^	37.2 ± 7.2
Carbohydrate (g)	189.6 ± 62.3
Carbohydrate (%) ^a^	41.7 ± 8.6
Starch (g)	100.7 ± 32.5
Starch (%) ^a^	22.2 ± 5.3
Total Sugar (g) ^b^	88.9 ± 38.0
Total sugar (%) ^a^	19.4 ± 6.1
Sucrose (g)	38.8 ± 21.7
Sucrose (%) ^a^	8.3 ± 3.5
Fructose (g)	18.9 ± 9.2
Fructose (%) ^a^	3.9 ± 1.7
Glucose (g)	17.6 ± 8.6
Glucose (%) ^a^	4.2 ± 2.2
Lactose (g)	11.3 ± 7.1
Lactose (%) ^a^	2.5 ± 1.5
Maltose (g)	2.8 ± 1.6
Maltose (%) ^a^	0.6 ± 0.3

All data were obtained from the four-day weighed food records and reported as mean ± SD. ^a^ Calculated as a % of total energy intake; ^b^ Total sugars include all mono- and di-saccharides. *n* = 41.

**Table 4:** Frequency of intake of sweet food categories.

Food Category	DFE ^a^
Fruit (e.g., bananas, apples, dried fruit)	1.8 [1.1, 3.4]
Baking/sweets (e.g., chocolates, biscuits, cakes)	1.2 [0.6, 1.7]
Beverages (e.g., fruit juice, soft drinks, fruit smoothies)	0.8 [0.3, 1.1]
Dairy (e.g., yoghurt, flavored milk, yoghurt drinks)	0.6 [0.2, 1.1]
Cereals (e.g., muesli, liquid breakfasts, cereals)	0.4 [0.1, 0.9]
Spreads/sweeteners (e.g., sugar, jam, honey/golden syrup)	0.3 [0.1, 0.9]
Vegetables (e.g., kumara, beetroot, pumpkin)	0.2 [0.2, 0.6]
Desserts (e.g., ice cream, custard, jelly)	0.2 [0.1, 0.2]
Total sweet food	7.1 ± 3.0

DFE: daily frequency equivalent. Total sweet food reported as mean ± SD. All other data reported as median [25, 75 percentiles]. ^a^ DFE score of 2—twice a day or more; 1—once per day; 0.71—4–6 times per week; 0.3—2–4 times per week; 0.14—once per week; 0.08—2–3 times per month; 0.03—Less than once a month; 0—never. *n* = 44.

**Table 5:** Liking scores of sweet beverages.

Sweet Beverage	Liking Score (mm)
Fruit Smoothie	24.2 ± 19.2
Cocktail	13.8 ± 27.1
Dessert wine/Cider	10.9 ± 29.3
Milk mixer	10.3 ± 22.4
Fruit Juice	8.8 ± 23.4
Iced coffee	1.8 ± 37.0
Flavored milk/Milkshakes	1.3 ± 30.1
Iced tea	−2.2 ± 29.9
Soft drink (regular)	−3.0 ± 28.3
Flavored water	−4.8 ± 24.5
Spirits	−6.0 ± 28.4
Soft drink (sugar free)	−6.6 ± 28.4
Yoghurt drink	−7.1 ± 28.5
Fruit drink	−8.2 ± 25.2
Cordial	−18.2 ± 22.1
Energy drinks	−23.2 ± 26.4

All data generated from the sweet beverage liking questionnaire and reported as mean ± SD. *n* = 44.

**Table 6:** Correlation coefficients of the relationship between sweet taste intensity and food intake.

	Sweet Taste Intensity			
Energy/Nutrients	125 mM	250 mM	500 mM	1000 mM
Total energy (kJ)	−0.19	−0.38 *	−0.36 *	−0.40 **
Protein (g)	−0.24	−0.1	−0.21	−0.20
Fat (g)	−0.01	−0.18	−0.07	−0.19
Carbohydrate (g)	−0.24	−0.42 **	−0.43 ***	−0.45 ***
Starch (g)	−0.28	−0.42 **	−0.40 **	−0.41 **
Total Sugars (g) ^a^	−0.22	−0.35 *	−0.36 *	−0.38 **
Sucrose (g)	−0.15	−0.27	−0.26	−0.29
Fructose (g)	−0.28	−0.28	−0.39 *	−0.37 *
Glucose (g)	−0.3	−0.34 *	−0.41 **	−0.41 **
Lactose (g)	−0.2	−0.3	−0.19	−0.18
Maltose (g)	0.04	−0.05	−0.22	−0.32 *

Correlation coefficients determined by Pearson’s correlation coefficient for parametric data and Spearman’s correlation coefficient for non-parametric data. ^a^ Total sugars include all mono- and di-saccharides. * *p* < 0.05, ** *p* < 0.01, *** *p* < 0.001. *n* = 41.

**Table 7:** Correlation coefficients of the relationship between sweet hedonic liking and food intake.

	Sweet Hedonic Liking			
Energy/Nutrients	125 mM	250 mM	500 mM	1000 mM
Total energy (kJ)	0.04	0.18	0.31 *	0.32 *
Protein (g)	−0.05	0.01	0.19	0.13
Fat (g)	−0.02	0.08	0.1	0.19
Carbohydrate (g)	0.003	0.13	0.34 *	0.36 *
Starch (g)	0.01	0.12	0.22	0.21
Total Sugars (g) ^a^	−0.01	0.11	0.37 *	0.41 **
Sucrose (g)	−0.05	0.08	0.23	0.29
Fructose (g)	−0.01	0.06	0.33 *	0.35 *
Glucose (g)	−0.06	0.02	0.39 *	0.42 **
Lactose (g)	0.01	0.12	0.31 *	0.27
Maltose (g)	0.08	0.14	0.40 **	0.46 **

Correlation coefficients determined by Pearson’s correlation coefficient for parametric data and Spearman’s correlation coefficient for non-parametric data. ^a^ Total sugars include all mono- and di-saccharides. * *p* < 0.05, ** *p* < 0.01. *n* = 41.
